# Screening for Diabetes Mellitus among Tuberculosis Patients: Findings from a Study at a Tertiary Hospital in Lusaka, Zambia

**DOI:** 10.1155/2018/3524926

**Published:** 2018-03-06

**Authors:** Sombo Fwoloshi, Lottie M. Hachaambwa, Kaseya O. Chiyeñu, Lameck Chirwa, Thijs W. Hoffman, Owen Ngalamika, Sarah Lou Bailey

**Affiliations:** ^1^Adult Infectious Diseases Center of Excellence, Department of Medicine, University Teaching Hospital, University of Zambia School of Medicine, Lusaka, Zambia; ^2^Department of Infectious Diseases, University of Maryland, Baltimore, MD, USA; ^3^Department of Medicine, Livingstone Central Hospital, Livingstone, Zambia; ^4^Dermatology and Venereology Section, University Teaching Hospital, University of Zambia School of Medicine, Lusaka, Zambia; ^5^TB Centre and Department of Clinical Research, London School of Hygiene and Tropical Medicine, London, UK

## Abstract

**Background:**

Diabetes mellitus (DM) is known to be associated with active tuberculosis (TB). Zambia is a low-income sub-Saharan African country with a high TB burden and increasing numbers of newly diagnosed DM patients.

**Materials and Methods:**

This was an observational study conducted at the University Teaching Hospital in Lusaka, Zambia, from October 2014 to February 2016. Adult patients with active TB were screened for DM.

**Results:**

A total of 127 individuals were enrolled in the study. Six patients (5%) were found to have diabetes. Of these, three had a prior diagnosis of diabetes and were on medication while three were newly diagnosed. Low education level was significantly associated with DM (*p*=0.001; 95% CI 0.001–0.148).

**Conclusion:**

The prevalence of DM among individuals with smear positive TB in our study population was similar to that of the general population in Zambia.

## 1. Introduction

One-third of the world is infected with tuberculosis (TB), with a large part of the TB burden falling on sub-Saharan Africa [1]. After initial infection with TB, the disease progresses to a dormant state, and only 10% of patients later develop active TB [2]. Progression to active disease is dependent on several factors, including the host immune response. Immunodeficiency states such as human immunodeficiency virus (HIV) infection, chronic disease, malnutrition, use of immunosuppressive medication, and diabetes mellitus (DM) are associated with higher rates of developing active TB disease [3].

DM is characterized by chronic hyperglycemia with contributions of absolute or relative insulin deficiency and increased insulin resistance in individually varying degrees. Individuals with overt symptoms are easily diagnosed with DM. On the other hand, it can be a challenge to make a diagnosis in an asymptomatic individual, especially in resource-constrained settings. The prevalence of DM is on the rise in low- and middle-income countries. The number of adults with DM in sub-Saharan Africa is predicted to rise from 14.7 million in 2011 to 28.0 million in 2030 [4].

Systematic reviews suggest that individuals with DM are around three times more likely to develop TB than the general population [5]. In addition, individuals with TB and DM have been seen to have a higher risk of TB relapse and a higher risk of death compared to individuals with TB alone [6]. Most of these studies have been conducted in non-African countries. It is therefore important for more of such studies to be conducted in Africa so as to determine the need for screening DM in TB patients and vice versa.

In Zambia, the ongoing epidemic of HIV combined with a predicted growing burden of DM may present a unique clinical and public health challenge. A study at the University Teaching Hospital (UTH) in Lusaka found sputum-producing DM patients to have sixfold increased odds of having culture-positive TB compared to patients without DM [7]. A population-based prevalence study carried out in Lusaka urban district found a combined prevalence of impaired glucose tolerance and diabetes of 4.0% [8], while another population-based survey [9] found a diabetes prevalence of 3.5% in adults from communities in several Zambian provinces. In the latter study, there was a hyperglycemia prevalence (random blood sugar (RBS) ≥11.1 mmol/L) of 1.5% [10]. The hyperglycemia was significantly associated with TB. Nevertheless, the population attributable fraction of TB to hyperglycemia was low at 0.99% (95% confidence interval 0.12–1.85%) [10].

Globally, there is growing concern over the dual epidemic of TB and DM [11, 12]. The World Health Organization and the International Union Against Tuberculosis and Lung Disease currently recommend screening all newly diagnosed TB patients for DM and to consider screening for TB in all patients with DM [13]. However, there is no nationwide screening policy in place in Zambia. To date, the association between DM and TB has not been studied extensively in Zambia. The aim of this study was to determine the prevalence of DM in TB patients at a tertiary referral hospital in Lusaka and hence determine whether the WHO screening policy is necessary for our hospital setting in Zambia.

## 2. Materials and Methods

This is an observational cross-sectional study. Participants were recruited consecutively from the outpatient chest clinic and the inpatient medical wards at the University Teaching Hospital (UTH) in Lusaka, Zambia. UTH is the national referral hospital for Zambia. However, it also has first-line outpatient and inpatient functions for patients in Lusaka. The study included all adult patients with a new diagnosis of smear positive pulmonary TB. Recruitment was based on routine sputum-smear results taken and processed in UTH. Only patients who had not yet started TB treatment or had been receiving TB treatment for less than seven days were included. Patients with extrapulmonary TB were excluded due to the lack of confirmatory diagnostic tests for extrapulmonary TB: in our setting, the vast majority of patients with extrapulmonary TB are given the diagnosis based on clinical and/or radiological evidence as it is not always possible to obtain and process deep tissue samples.

### 2.1. Study Procedures

All the participants were treated with the standard TB treatment regimen, in accordance with the Zambian National Treatment Guidelines for Tuberculosis [14]. At the time of the study, the recommended TB treatment regimen included pyrazinamide, ethambutol, isoniazid, and rifampicin. Streptomycin was added for re-treatment regimens. All regimens are given as a fixed dose combination, with dosage based on weight bands.

Fasting blood sugar (FBS) was checked in all the patients using a point of care glucometer. Participants who were known to have diabetes or with an FBS of more than or equal to 7 mmol/L were considered to have diabetes.

At recruitment, participant data were collected during a participant interview by one of the study investigators using a previously piloted questionnaire. The data included age, gender, weight and height, socioeconomic status, HIV status, and other possible immunodeficient states like malignancies or immunosuppressive drugs (such as steroids).

Data collected from participants were entered directly onto a preprogrammed Personal Digital Assistant (PDA) at the time of data collection and were then downloaded into a secure database specifically designed for the study and with appropriate privacy safeguards. Body mass index (BMI) was calculated from height and weight data.

### 2.2. Data Analysis

The collected data were analyzed using Stata, Version 12. Descriptive statistics were used to analyze the baseline characteristics, univariate logistic regression to assess crude associations by determining crude odds ratios, and multivariate logistic regression to assess adjusted associations by determining adjusted odds ratios. Variables that were found to have good evidence of association with diabetes mellitus on univariate analysis were included in the final multivariate model.

### 2.3. Ethics

The study followed all procedures in accordance with the Declaration of Helsinki (2013 version). Formal written informed consent was required of every participant. Each participant received an information sheet detailing the aims and methods of the study prior to being asked for consent. Strict confidentiality was maintained throughout the study. Ethical approval was secured prior to study commencement from ERES Converge Institutional Review Board.

## 3. Results

**Figure 1:**
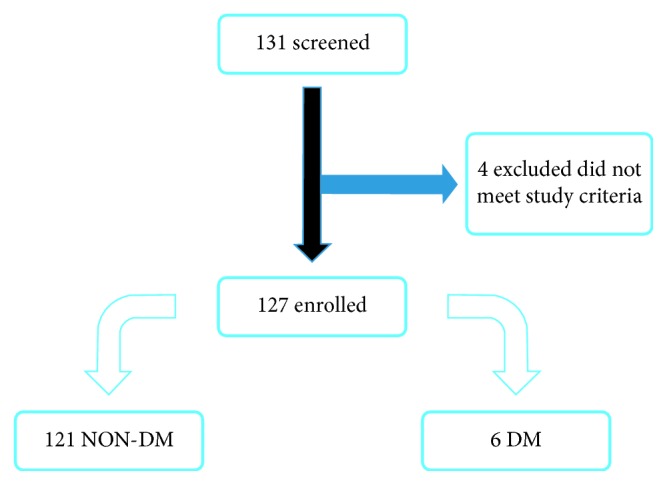
Recruitment and enrollment process.

One hundred thirty-one participants were screened. One participant was excluded as a sputum smear result was not available, and three participants were excluded because they were not in a fasted state for the FBS measurement. One hundred twenty-seven participants were finally enrolled into the study.  Figure 1 illustrates the recruitment and enrollment process. One hundred six participants were recruited from the outpatient chest clinic, and the remainder from inpatient wards.

Of the 127 participants, six (4.7%) were considered to have diabetes, defined as per the study protocol. Of these, three were previously known to have diabetes and were on treatment while the other three were newly diagnosed.  Table 1 summarizes the baseline demographic and clinical characteristics. No significant differences in age were observed between the two groups. The mean age for the participants with DM was 33 years versus 37 years for the participants without DM. However, among the participants with DM, there were none over 50 years of age, while among the participants without diabetes, 17 (14.1%) were between 51 and 85 years. All participants with DM knew their HIV status while almost half of the nondiabetics (42.2%) did not know or were unwilling to disclose their HIV status. The gaps in the table are mainly due to a lack of study power to do the analyses due to a low prevalence of diabetes.

The median FBS for the participants without DM was 5.2 (IQR 5.9–4.6) versus 8.1 (IQR 7.0–10.8) for participants with DM. All measured FBS values were less than 10 mmol/L except for one individual who had an FBS of 19.5 mmol/L. There were more females than males with DM in our study. Education was strongly associated with DM both on univariate and multivariate analyses; individuals with no formal education were more likely to have DM than individuals with any level of education. All diabetics in our study had either a normal or low BMI.

## 4. Discussion

In this study conducted at a tertiary referral hospital in Lusaka, Zambia, only 4.7% of individuals with TB were found to have DM. Half of these (2.3% of total) were newly diagnosed with DM through participation in this study. This is considerably lower than the reported prevalence of DM in similar cohorts of TB patients in sub-Saharan Africa [15–18]. The prevalence of DM in our cohort is similar to the estimated overall prevalence of DM in Lusaka [9]. This suggests that DM is not a driver of the TB epidemic in Zambia. A previous study at the same hospital showed that TB was highly prevalent in patients diagnosed with DM [7]. This implies that case finding of DM in TB patients might not be useful in this resource-constrained setting, whereas case finding of TB in DM patients may be more useful.

Individuals without any formal education had a statistically significant increased likelihood of having DM than those with a history of formal education. As education level is usually associated with low incomes in a country like Zambia, this finding is in line with other studies that have observed that low education is associated with a higher prevalence of DM [19]. However, the numbers of our study participants were too low to conclude.

There is a very strong association between obesity and the risk for development of type 2 diabetes [20]. 11 (9.8%) of our study participants were either overweight or obese. However, none of these were found to be diabetic, and all diabetics were either of normal weight or underweight. This may be explained by the weight loss attributed to chronic infections such as HIV and TB. Furthermore, it is not known whether our newly diagnosed DM study participants merely had transient hyperglycemia or whether it was type 1 and not type 2 diabetes, all the known diabetics were well controlled on oral hypoglycemics except for one individual. A lack of study power makes interpretation of associations very difficult. Isoniazid and rifampicin were ruled out as possible causes of hyperglycemia because our study participants were newly diagnosed TB patients that were not on any TB treatment or were on TB drugs for less than 7 days.

As expected, we had more males enrolled in the study than females, as TB is more common among males [3]. This study had more females than males in the DM group. The few other prior studies that have been conducted in Africa have not shown any gender bias in DM prevalence [21].

The results of our study must be interpreted with caution due to the lack of study power resulting from the low prevalence of diabetes that we found among our participants. Although the prevalence of diabetes found in our study participants, who all had TB, was similar to the reported prevalence found in other studies conducted in Lusaka and Zambia, it is worth noting that most of our participants were in the age range of 18 to 35 years, while, in general, diabetes tends to affect people who are older. Considering the young age of diabetics in our study, it may have been useful to measure the serum anti-GAD antibodies and/or c-peptide to rule out type 1 diabetes. However, this was not possible in our study due to the lack of reagents to do those tests in our hospital laboratory.

The HIV status was unknown for a significant proportion of study participants. However, more than half of the participants had a known status, and of these, more than half were HIV negative. This is consistent with the fact that HIV-negative individuals are more likely to have a positive smear for TB than their HIV-positive counterparts [22, 23]. However, HIV infection is known to increase the risk of reactivation of latent TB [24]. Only one of the DM participants was HIV positive.

Further research is needed to characterize the pattern of disease in TB-DM comorbid individuals in Zambia and the rest of Africa. Given the clear association between DM and TB elsewhere in the world, as Africa develops, deliberate efforts should be made by the Ministries of Health and other stakeholders to facilitate active surveillance of noncommunicable diseases like DM among individuals who present with communicable diseases like TB and vice versa as overlap of these could increase in the future. Thus, through such implementations, a database can be created which can be used as a basis for further research.

## 5. Conclusion

Overall, the prevalence of DM among individuals with smear-positive TB was similar to that of the general population in Zambia. This study did not provide evidence to support the introduction of diabetes screening for all TB patients in our setting, and so rather, screening should continue to be done on a case-by-case basis. However, given the clear association seen between DM and TB elsewhere in the world and as the incidence of DM is expected to keep rising in low- and middle-income countries, there will still be need to duplicate similar studies in the future.

## Figures and Tables

**Table 1:** Baseline characteristics of 127 pulmonary TB patients and odds ratios for DM.

Characteristic	Participants without diabetes, *N* (%)	Participants with diabetes, *N* (%)	Univariate OR (95% CI)	*p* value	Multivariate OR (95% CI)	*p* value
*Total*	121 (95.3)	6 (4.7)	—	—	—	—
*Age*					—	—
18–35	69 (57.0)	3 (50.0)	1			
36–50	35 (28.9)	3 (50.0)	1.97 (0.38–10.28)	0.420		
51–85	17 (14.0)	0 (0.0)				
*Sex*						
Male	38 (31.4)	1 (16.7)	1		1	
Female	83 (68.6)	5 (83.3)	10.92 (1.23–96.72)	0.032	9.99 (0.66–151.3)	0.097
*HIV infection*					—	—
No	45 (37.2)	5 (83.3)	1			
Yes	25 (20.7)	1 (16.7)	0.36 (0.04–3.26)	0.363		
Missing	51 (42.2)					
*Fasting blood sugar, median (IQR)*	5.2 (4.6–5.9)	8.1 (7.0–10.8)	—	—	—	—
*Education*						
No formal education	2 (1.7)	4 (66.7)	1		1	
Primary	39 (33.3)	1 (16.7)	0.01 (0.00–0.18)	0.001	0.01 (0.00–0.22)	0.003
Secondary	46 (39.3)	1 (16.7)		0.001		0.002
Tertiary	30 (25.6)	0 (0.0)	0.01 (0.00–0.15)		0.01 (0.00–0.21)	
*Smoked*			—	—	—	—
No	108 (92.3)	2 (33.3)				
Yes	9 (7.7)	0 (0.0)				
*Body mass index*					—	—
Under weight (<18.5)	31 (27.7)	1 (16.7)	1			
Healthy weight (18.5–24.9)	70 (62.5)	5 (83.3)	2.11 (0.25–19.75)	0.476		
Overweight (25–29.9)	9 (8.0)	0 (0.0)				
Obese (>30)	2 (1.8)	0 (0.0)				
*Abdominal circumference, mean (SD)*	64.3 (10.0)	64.5 (13.9)	—	—	—	—
*Location of recruitment*					—	—
Inpatient	20 (16.5)	1 (16.7)	1			
Outpatient	101 (83.5)	5 (83.3)	0.99 (0.11–8.94)	0.993		

IQR: interquartile range; SD: standard deviation; OR: odds ratio; 95% CI: 95% confidence interval. Multivariable analyses were adjusted for age.

## References

[1] WHO (2016). *Global Tuberculosis Report 2016*.

[2] Kondratieva T., Azhikina T., Nikonenko B., Kaprelyants A., Apt A. (2014). Latent tuberculosis infection: what we know about its genetic control?. *Tuberculosis*.

[3] Mehta J. B., Dutt A. K. (2016). Epidemiology and host factors. *Microbiology Spectrum*.

[4] Whiting D. R., Guariguata L., Weil C., Shaw J. (2011). IDF diabetes atlas: global estimates of the prevalence of diabetes for 2011 and 2030. *Diabetes Research and Clinical Practice*.

[5] Jeon C. Y., Murray M. B. (2008). Diabetes mellitus increases the risk of active tuberculosis: a systematic review of 13 observational studies. *PLoS Medicine*.

[6] Baker M. A., Harries A. D., Jeon C. Y. (2011). The impact of diabetes on tuberculosis treatment outcomes: a systematic review. *BMC Medicine*.

[7] Bates M., O’Grady J., Mwaba P. (2012). Evaluation of the burden of unsuspected pulmonary tuberculosis and co-morbidity with non-communicable diseases in sputum producing adult inpatients. *PLoS One*.

[8] Nsakashalo-Senkwe M., Siziya S., Goma F. M., Songolo P., Mukonka V., Babaniyi O. (2011). Combined prevalence of impaired glucose level or diabetes and its correlates in Lusaka urban district, Zambia: a population based survey. *International Archives of Medicine*.

[9] Bailey S. L., Ayles H., Beyers N. (2016). Diabetes mellitus in Zambia and the Western Cape province of South Africa: prevalence, risk factors, diagnosis and management. *Diabetes Research and Clinical Practice*.

[10] Bailey S. L., Ayles H., Beyers N. (2016). The association of hyperglycaemia with prevalent tuberculosis: a population-based cross-sectional study. *BMC Infectious Diseases*.

[11] Dooley K. E., Chaisson R. E. (2009). Tuberculosis and diabetes mellitus: convergence of two epidemics. *Lancet Infectious Diseases*.

[12] Bailey S. L., Grant P. (2011). ‘The tubercular diabetic’: the impact of diabetes mellitus on tuberculosis and its threat to global tuberculosis control. *Clinical Medicine*.

[13] I. U. A. T. a. L. D. WHO (2011). *Collaborative Framework for Care and Control of Tuberculosis and Diabetes*.

[14] T. Z. N. T. a. L. Programme (2008). *TB Manual*.

[15] Alladin B., Mack S., Singh A. (2011). Tuberculosis and diabetes in Guyana. *International Journal of Infectious Diseases*.

[16] Faurholt-Jepsen D., Range N., Praygod G. (2011). Diabetes is a risk factor for pulmonary tuberculosis: a case-control study from Mwanza, Tanzania. *PLoS One*.

[17] Kibirige D., Ssekitoleko R., Mutebi E., Worodria W. (2013). Overt diabetes mellitus among newly diagnosed Ugandan tuberculosis patients: a cross sectional study. *BMC Infectious Diseases*.

[18] Workneh M. H., Bjune G. A., Yimer S. A. (2016). Prevalence and associated factors of diabetes mellitus among tuberculosis patients in South-Eastern Amhara Region, Ethiopia: a cross sectional study. *PLoS One*.

[19] Rabi D. M., Edwards A. L., Southern D. A. (2006). Association of socio-economic status with diabetes prevalence and utilization of diabetes care services. *BMC Health Services Research*.

[20] Whitmore C. (2010). Type 2 diabetes and obesity in adults. *British Journal of Nursing*.

[21] Hilawe E. H., Yatsuya H., Kawaguchi L., Aoyama A. (2013). Differences by sex in the prevalence of diabetes mellitus, impaired fasting glycaemia and impaired glucose tolerance in sub-Saharan Africa: a systematic review and meta-analysis. *Bulletin of the World Health Organization*.

[22] Elliott A. M., Namaambo K., Allen B. W. (1993). Negative sputum smear results in HIV-positive patients with pulmonary tuberculosis in Lusaka, Zambia. *Tubercle and Lung Disease*.

[23] Getahun H., Harrington M., O’Brien R. (2007). Diagnosis of smear-negative pulmonary tuberculosis in people with HIV infection or AIDS in resource-constrained settings: informing urgent policy changes. *The Lancet*.

[24] Brassard P., Hottes T. S., Lalonde R. G. (2009). Tuberculosis screening and active tuberculosis among HIV-infected persons in a Canadian tertiary care centre. *Canadian Journal of Infectious Diseases and Medical Microbiology*.

